# Multisite Radiotherapy Combined With Tislelizumab for Metastatic Castration-Resistant Prostate Cancer With Second-Line and Above Therapy Failure: Study Protocol for an Open-Label, Single-Arm, Phase Ib/II Study

**DOI:** 10.3389/fonc.2022.888707

**Published:** 2022-07-07

**Authors:** Ke Cheng, Yuqing Wang, Ye Chen, Jingjie Zhu, Xiaohui Qi, Yachen Wang, Yanqiu Zou, Qiuhan Lu, Zhiping Li

**Affiliations:** ^1^ Department of Abdominal Oncology, West China Hospital, Sichuan University, Chengdu, China; ^2^ State Key Laboratory of Biotherapy, West China Hospital, Sichuan University, Chengdu, China; ^3^ West China School of Medicine, West China Hospital, Sichuan University, Chengdu, China; ^4^ West China School of Public Health, Sichuan University, Chengdu, China; ^5^ Laboratory of Clinical Pharmacy and Adverse Drug Reaction, West China Hospital, Sichuan University, Chengdu, China; ^6^ Department of Radiotherapy, Cancer Center, West China Hospital, Sichuan University, Chengdu, China

**Keywords:** metastatic castration-resistant prostate cancer (mCRPC), tislelizumab, PD-1 monoclonal antibodies, combination therapy, study protocol, multisite radiotherapy

## Abstract

**Background:**

Tislelizumab combined with radiotherapy as a salvage treatment for patients with end-stage metastatic castration-resistant prostate cancer (mCRPC) is not reported. This study aimed to describe a protocol to evaluate the safety and efficacy of multisite radiotherapy combined with tislelizumab as a salvage therapy for mCRPC in patients who had at least one second-line treatment failure.

**Methods:**

The study included patients with mCRPC who had at least one lesion suitable for radiotherapy and failed androgen deprivation therapy (ADT), followed by at least one novel second-line endocrine therapy. All patients received tislelizumab monotherapy induction therapy for two cycles, then combined with multisite radiotherapy for one cycle, followed by tislelizumab maintenance therapy, until either disease progressed or the patient developed unacceptable toxicity. Radiation methods and lesions were individually selected according to the specified protocol. Primary endpoints included safety and objective response rate. Secondary endpoints included prostate-specific antigen (PSA) response rate, disease control rate, overall survival, radiographic progression-free survival (rPFS), and biochemical progression-free survival (bPFS). Furthermore, the exploratory endpoints included the identification of the predictive biomarkers and exploration of the correlation between biomarkers and the tumor response to the combined regimen.

**Discussion:**

This study included three treatment stages to evaluate the efficacy of immunotherapy and the combination of immunotherapy and radiotherapy for patients with mCRPC who have had at least second-line treatment failure. Additionally, radiation-related and immune-related early and late toxicities were determined, respectively. Furthermore, the study also aimed to identify the predictive biomarkers associated with immunotherapy for treating mCRPC.

**Trial Registration:**

https://www.chictr.org.cn/showproj.aspx?proj=126359, identifier ChiCTR2100046212.

## Introduction

Prostate cancer (PCa) is the world’s second leading cause of cancer-related mortality in men (1). China has the sixth-highest rate of incidence and mortality due to PCa (2). Androgen deprivation therapy (ADT) is one of the most important therapies for patients with hormone-sensitive PCa. Unfortunately, most patients with PCa eventually develop metastatic castration-resistant prostate cancer (mCRPC) within 2–3 years of undergoing ADT (3, 4). Currently, multiple approved therapies can prolong the survival of patients with mCPRC, including new-generation hormone drugs, such as abiraterone and enzalutamide, and chemotherapeutic drugs, such as docetaxel and cabazitaxel, targeted therapy drugs, and immunotherapy drugs (Sipuleucel-T) (5). However, despite the efficacy of these drugs, cancer cells inevitably develop resistance to them (6). Once patients fail the second-line endocrine therapy, there is a lack of a standard treatment model for subsequent treatments. A clinical trial suggested that a common subset of mCRPC, characterized by defects in DNA repair, could be treated using the poly (ADP-ribose) polymerase (PARP) inhibitor olaparib in patients with mCRPC who had developed resistance to standard treatments (7, 8). However, this subset only accounted for approximately 11.8% of all sporadic mCRPC (2). Most patients could not achieve durable responses with available treatments. Thus, it was a fundamental requirement to identify novel strategies to improve the survival of patients with mCRPC after the failure of second-line endocrine therapy.

The introduction of immunotherapy-targeted programmed death protein 1 (PD-1) and programmed cell death-ligand 1 (PD-L1) has altered the treatment paradigm for various types of malignancies. Unfortunately, immunotherapy has only shown modest efficacy against PCa (9). As per the results of two recently published clinical studies, two anti-PD-1 antibodies, pembrolizumab and atezolizumab were well tolerated and safe in patients with mCRPC. However, complete response (CR) was achieved in only a few patients (10, 11). Tislelizumab, an investigational anti-PD-1 antibody, has been shown to be significantly efficacious in (85.7% objective response rate (ORR)) patients with relapsed/refractory classical Hodgkin’s lymphoma (12). A recently published research demonstrated that tislelizumab showed substantial clinical benefits and an acceptable safety profile in patients with urothelial carcinoma (13). Another study found that tislelizumab had disease stabilization capacity for various tumor types and in patients who had undergone different types of long-term treatments (14). Thus, it was speculated that tislelizumab might act as an effective salvage treatment strategy to improve the outcomes in patients with mCRPC. However, due to the “cold tumor” characteristics of PCa, the response to immunotherapy in PCa might not be as strong compared with other tumors (1). Thus, a combination of immunotherapy and other current treatment strategies, such as chemotherapy, targeted therapy, and radiotherapy (RT), could improve the immune response to “cold tumors.” Previous studies have reported that ipilimumab monotherapy or the addition of atezolizumab to enzalutamide for treating patients with mCRPC could not provide a satisfactory primary endpoint for overall survival (OS) (15, 16). However, using ipilimumab plus RT showed improved outcomes compared with placebo plus RT in patients with postdocetaxel mCRPC (17). Consequently, RT could be a promising strategy for the synergistic enhancement of immunotherapeutic efficacy.

Numerous clinical trials have supported the use of RT in the modification of antitumor immune responses, enhanced expression of antigens on the surface of tumor cells, as well as tumor antigen crosspresentation in the draining lymph nodes, directly resulting in the activation and proliferation of tumor-specific cytotoxic T cells (18–21). Consequently, this might result in a modified tumor microenvironment along with the expansion of immunotherapeutic capacity (22, 23). Multisite stereotactic body radiotherapy (SBRT) has emerged as an altering paradigm for treating solid metastatic tumors (24). A phase I study indicated that multisite SBRT combined with pembrolizumab for solid metastatic tumors was well tolerated with acceptable levels of toxicity (25). However, there is a scarcity of sufficient studies examining the therapeutic effects of combined anti-PD-1 and multisite SBRT in mCRPC treatment, necessitating further research. Another phase II trial demonstrated that avelumab combined with SBRT exhibited elevated activity and acceptable toxicity in treatment-refractory mCRPC (26). Although a study reported that SBRT with a few fractionations was the best choice for the abscopal effect (27), an appropriate RT technique should be chosen based on the symptoms and condition of the patient with mCRPC. Previous studies have demonstrated that low-dose radiation (e.g., doses below 3 Gy) could promote immune cell infiltration into the stroma and the tumor bed of distant tumors, resulting in an improved rate of the systemic response to metastatic disease (28).

Furthermore, low-dose radiotherapy against established metastases has also been shown to significantly enhance the abscopal response to hypofractionated RT plus immune checkpoint inhibitors (29). Low-dose radiation also carries the potential to amplify the antitumor immune effects. Another study suggested that low-dose radiation (a maximum dose of 8–10 Gy/fraction) could induce interferon signaling, resulting in RT-induced abscopal outcomes (30). Several studies also indicated that multiple dose-fractionation schedules of RT resulted in an enhanced abscopal effect compared to a single dose (31, 32). However, further research is required to explore whether the reported doses of RT would exhibit the activation impact in combination with immunotherapy. Thus, combination trials of immunotherapy and RT could be designed to optimize the choice of optimal dose and fractionation.

Here, we aimed to analyze the safety and efficacy of multisite radiotherapy combined with tislelizumab for patients with mCRPC who have experienced failure of at least one second-line treatment. Additionally, we planned to explore the predictive biomarkers of the efficacy of this combined regimen to facilitate clinical studies.

## Materials and Methods

### Study Design

This study is an open-label, single-arm, phase Ib/II prospective study including patients with mCRPC who experienced disease progression after treatment with ADT and had at least one second-line endocrine therapy failure (abiraterone acetate or enzalutamide). This study includes 48 patients, with the entire study (treatment and follow-up phases) lasting approximately 36 months; the maximum duration of tislelizumab treatment has been limited to 2 years. This study protocol has been approved by the Ethics Review Committee of West China Hospital, Sichuan University (Ethical approval number: 2021203). Written informed consent was obtained from all patients. The study has been registered on the Chinese Clinical Trials Registry (Chictr.org.cn) with registration number: ChiCTR2100046212.

### Eligibility Criteria

All patients who conformed to the inclusion criteria were included (**Table 1**). Additionally, patients would be able to withdraw from the study if they experience progression of disease (PD), elevated levels of toxicities, are lost to follow-up, die, undergo protocol violation, concomitant disease, or based on the investigator’s decision.

**Table 1:** The key inclusion and exclusion criteria of this study.

**Inclusion criteria**
1. Patients with incurable metastatic or unresectable prostate cancer, which was confirmed by histopathology and/or cytology (including postoperative recurrence and metastasis) without neuroendocrine differentiation or small cell features. 2. Patients who failed ADT therapy combined with at least one novel endocrine therapy (including enzalutamide, abiraterone, apalutamide, and so on, while not including bicalutamide and flutamide) or failed ADT therapy followed by at least one novel endocrine therapy. 3. Patients with hormone-sensitive prostate cancer (HSPC) who had not received ADT therapy combined with a docetaxel regimen, or patients who required or were unable to tolerate or refused docetaxel regimen chemotherapy after diagnosis of CRPC. 4. Patients with mCRPC with DNA-HRR gene mutation who had not received PARP inhibitor therapy, who had refused PARP inhibitor therapy, or had a contraindication to PARP inhibitor therapy. 5. Disease progression was recorded in patients (disease progression was defined as one or more of the following 3 events) in the 6 months prior to enrolment: 1. PSA progression: elevated PSA levels were measured at least thrice with an interval of ≥1 week, and the PSA value was expected to be ≥2 ng/ml each time. 2. For patients without PSA progression, imaging (RECIST 1.1) assessed the presence of soft tissue or bone metastatic lesion progression. 3. PCWG2-defined progression of bone lesions, i.e., two or more new lesions found on bone scan. 4. Patients with clinical evidence of distant metastatic disease (based on bone scan, CT/MRI). 5. For patients currently on continuous ADT therapy, serum total testosterone was required to be <50 ng/dl. 6. The ECOG PS ≤2. 7. Patients with expected survival time >6 months. 8. Patients with adequate organ and bone marrow function. Laboratory tests should meet the following criteria:
1. Routine blood test: Hb ≥90 g/L (no blood transfusion within last 14 days); ANC ≥1.5 × 10^9^/L; PLT ≥100 × 10^9^/L 2. Biochemical tests: CR ≤1.5 × ULN or CRCL ≥60 mL/min when serum creatinine >1.5 × ULN of subjects; Bilirubin Bil ≤1.5 × ULN; ALT and AST ≤2.5 × ULN (subjects with liver metastasis ≤5 × ULN) 3. Coagulation function: the INR <1.5. 9. Reproductive men should use an appropriate method of contraception for a period of 120 days from the first study drug administration to the last study drug administration.
**Exclusion criteria**
1. Patients who had not recovered from the toxicity induced by the original treatment regimen and still had toxicity reactions >grade 1 before enrolment. 2. Patients who participated in clinical trials of other drugs within the last 1 month. 3. Patients who had been diagnosed with immunodeficiency or were receiving systemic steroid therapy or any other form of immunosuppressive therapy 7 days prior to study initiation. If patients had to receive systemic steroid therapy (e.g., prednisone) before the start of immunotherapy, the maximum allowed dose of prednisone was 10 mg/day, else they were excluded. 4. Patients who had used or were using a FAK inhibitor or anti-PD-1, anti-PD-L1, anti-PD-L2, anti-CD137, or anti-cytotoxic T-lymphocyte-associated antigen 4 (CTLA-4) antibody (including ipilimumab or any other antibody or drug targeting the T-cell costimulatory or checkpoint pathway) within 4 weeks prior to study initiation. 5. Patients who had a history of other malignant tumors (except for basal cell carcinoma or orthotopic cervical cancer) within the last 5 years. 6. Patients with known or suspected new BMs: subjects with signs or symptoms suggestive of BMs were not allowed to participate in the study unless BMs had been ruled out by CT or MRI. However, subjects with controlled BMs (no radioactivity progression for at least 4 weeks after radiotherapy and/or no neurological symptoms or signs after surgical resection) were enrolled. 7. Patients who had an active autoimmune disease requiring systemic treatment (e.g., use of disease modifiers, corticosteroids, or immunosuppressive drugs) within the past 2 years. 8. Patients who had interstitial pulmonary disease and/or present (noninfectious) pneumonia requiring continued steroid therapy. 9. Patients who had combined active infection requiring systemic treatment. 10. Patients who had a history of epilepsy or were taking drugs that caused epilepsy or had a history of severe central nervous system diseases. 11. Patients who had severe cardiovascular disease, previous myocardial infarction or arterial thrombosis, unstable angina pectoris, or heart failure with clinical symptoms in the past 6 months. 12. Patients who had serious, uncontrolled medical disorders or active infections that could impair their ability to receive treatment as prescribed in the protocol, including but not limited to HIV positive and active tuberculosis. 13. Researchers considered the patients were inappropriate to participate.

ADT, androgen deprivation therapy; HSPC, hormone-sensitive prostate cancer; CRPC, castration-resistant prostate cancer; mCRPC, metastatic castration-resistant prostate cancer; PARP, poly (ADP-ribose) polymerase; PSA, prostate-specific antigen; RECIST 1.1, Response Evaluation Criteria in Solid Tumors; PCWG2, Prostate Cancer Working Group; ECOG PS, Eastern Cooperative Oncology Group Performance Status; Hb, hemoglobin; ANC, neutrophils absolute value; PLT, platelet; CR, serum creatinine; CRCL, creatinine clearance rate; AST, aspartate aminotransferase; INR, international standardized ratio; ULN, upper limit of normal; BMs, brain metastases; CT, computed tomography; MRI, magnetic resonance imaging; HIV, human immunodeficiency virus.

PD was assessed *via* imaging (CT/MRI/bone scan) as per Prostate Cancer Working Group 3 (PCWG3)–modified RECIST v1.1, a revised version in PCWG3 based on PCWG2.

### Procedures

**Figure 1:**
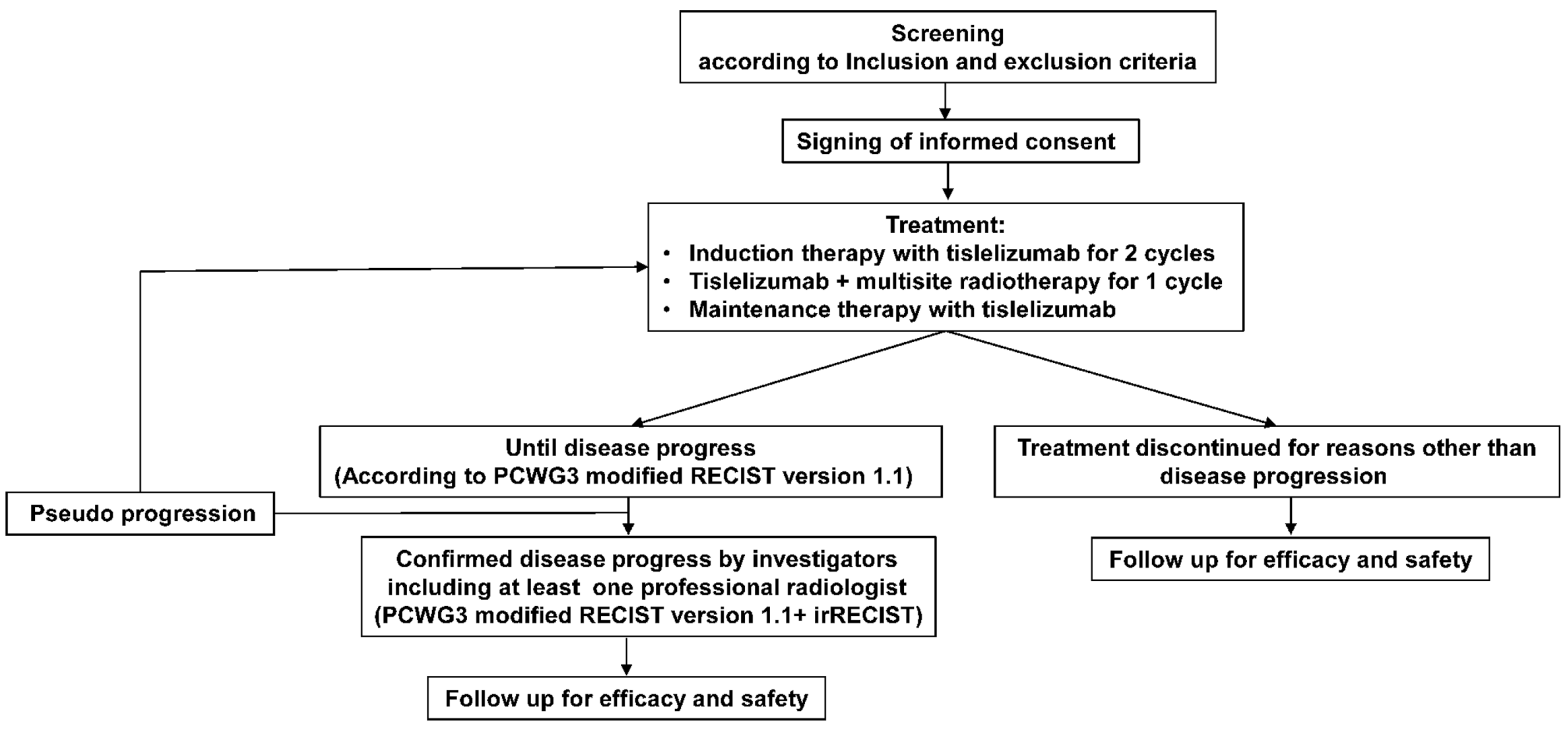
Flowchart of this study. PCWG3, Prostate Cancer Working Group 3; RECIST version 1.1, Response Evaluation Criteria in Solid Tumors version 1.1; irRECIST, Immune-related Response Evaluation Criteria in Solid Tumors.

**Figure 1** summarizes the execution outline of this study. Even if patients discontinue treatment due to disease progression, toxicity, or any other reason, they were followed up every 3 months after the end of treatment.

### Screening

The enrolled patients were screened within 2 weeks of the initiation of treatment. The following necessary procedures were performed during the screening: a collection of demographic and medical history, physical examination, estimation of PS ECOG, diagnosis and staging of the primary tumor, laboratory examination (blood, liver, kidney, heart, and thyroid function examinations), and imaging analysis. Finally, enrolled patients were required to sign a written informed consent.

### Treatment

**Figure 2:**
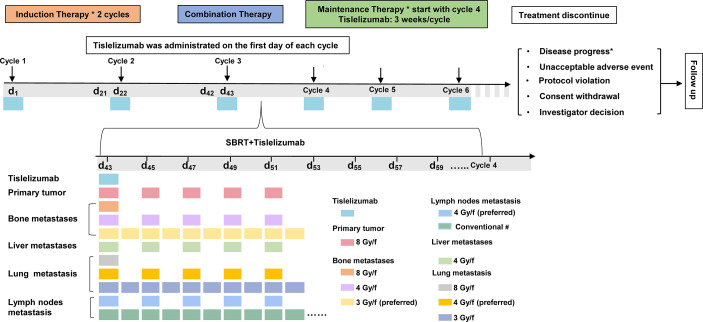
Flowchart of the treatment protocol. The therapeutic scheme has been divided into four phases: Induction phase, where patients were scheduled to receive the tislelizumab every 3 weeks (21-day cycle) for two cycles; Combination phase, where patients were scheduled to receive the SBRT (once every 2 days during one cycle) combined with tislelizumab (on day 1 every cycle); and Consolidation and maintenance phases, at 14 days after completing synchronous radiation, where patients were scheduled to receive tislelizumab alone on day 1 of a 21-day cycle until treatment was discontinued. During the follow-up, observing this study’s safety and clinical efficacy were observed. Abbreviations: PD-1, programmed cell death-1; SBRT, stereotactic body radiation therapy. The asterisk indicates that disease progression was confirmed according to the modified RECIST 1.1 of PCWG3. The number sign indicates that patients would receive conventional radiotherapy with 2 Gy every day up to a total dose of 40 or 50 Gy if the surrounding critical organs were at risk around lymph nodes, such as the duodenum, small intestine, and colon.

**Figure 2** shows the therapeutic scheme, which has been divided into three phases, including induction therapy (phase 1), combination therapy (phase 2), and maintenance therapy (phase 3).

In phase 1, patients received 1-h intravenous tislelizumab (provided by BeiGene, Beijing, China) at 200 mg every 3 weeks for two cycles. As described in **Table 2**, tislelizumab was suspended or terminated in the case of severe adverse events.

**Table 2:** Dose adjustment protocol for tislelizumab.

Adverse events	Severity	Dose adjustment
Pneumonia	Grade 2 of pneumonia	Dose interruption^a^
	Recurrent grade 2 of pneumonia, grade 3/4 of pneumonia	Permanent discontinuation
Diarrhea/enterocolitis	Grade 2/3 of diarrhea or enterocolitis	Dose interruption^a^
	Grade 4 of diarrhea or enterocolitis	Permanent discontinuation
Dermatitis	Grade 3 of dermatitis	Dose interruption^a^
	Grade 4 of dermatitis	Permanent discontinuation
Hepatitis	Grade 2 AST, ALT, or TBIL was increased in patients with normal baseline ALT, AST, or TBI; patients with AST, ALT, or TBIL above 50% (achieve level 2 requirements) and the duration <7 days	Permanent discontinuation^a^
	Grade 3/4 AST, ALT, or TBIL was increased in patients with normal baseline ALT, AST, or TBI; patients with AST, ALT, or TBIL above 50% (achieve level 3/4 requirements) and the duration ≥7 days	Permanent discontinuation
Inflammatory of the pituitary gland	Grade 2 of the pituitary gland inflammatory	Dose interruption^b^
	Grade 3/4 of the pituitary gland inflammatory	Permanent discontinuation
Adrenocortical dysfunction	Grade 2 of the adrenocortical dysfunction	Dose interruption^b^
	Grade 3/4 of the adrenocortical dysfunction adrenocortical dysfunction	Permanent discontinuation
Hyperthyroidism	Grade 3/4 of the hyperthyroidism	Permanent discontinuation
Type I diabetes	Grade 3 of hyperglycemia	Dose interruption^b^
	Grade 4 of hyperglycemia	Permanent discontinuation
Renal insufficiency	Grade 2/3 CR increased	Dose interruption^a^
	Grade 4 CR increased	Permanent discontinuation
Neurotoxicity	Grade 2 neurotoxicity	Dose interruption^a^
	Grade 3/4 neurotoxicity	Permanent discontinuation
Other AE	The first time occurs for other level 3 AEs	Dose interruption^b^
	The same level 3 AE occurs a second time	Permanent discontinuation
	Grade 3 AE cannot be reduced to baseline level for 0–2 within 7 days or returned to baseline level for 0–1 within 14 days	Permanent discontinuation
	Grade 4 AE	Permanent discontinuation^c^

The maximum duration of dose interruptions was 12 weeks. However, if patients were unable to tolerate tislelizumab, then it was permanently discontinued, and patients were followed, except for the following two conditions (1): Tislelizumab was interrupted for more than 12 weeks due to a dose reduction of glucocorticoids (glucocorticoid was used for immune-related AE treatment). The investigator and sponsor decided whether patients would continue to receive tislelizumab treatment. However, during dose interruption, the imaging tests, which were used for efficacy assessment, were conducted as planned (2). Tislelizumab was interrupted for more than 12 weeks due to treatment for AE that was unrelated to tislelizumab. The investigator and sponsor decided whether patients would continue to receive tislelizumab treatment. However, during dose interruption, the imaging tests, which were used for efficacy assessment, were conducted as planned. If the toxicity returned to grade ≤1 or baseline, and the ECOG PS ≤1, patients could continue to receive tislelizumab treatment. Notice that in the stage 1 study, if 14/29 patients stopped the treatment because of SAEs, the study was stopped early.

a Dosing could be resumed once the symptoms improve to grade 0–1 or baseline.

b Dosing could be resumed for patients who had pituitary or adrenocortical insufficiency, hypothyroidism, and type 1 diabetes, once the diseases were adequately controlled using physiological hormones.

c Investigator decided to terminate medicine for abnormal results in grade 4.

In phase 2, patients received tislelizumab combined with multisite RT for one cycle. During this phase, tislelizumab was administered at 200 mg once every 3 weeks; the schedule for multisite radiotherapy is presented in **Figure 2**. RT was performed using the intension-modulated radiotherapy (IMRT) technique under computed tomographic (CT) localization at 6MV-X rays. SBRT was recommended as the method of choice for RT.

The RT was administered to one or three disease sites, selected based on a prioritization order **(** **Table 3** **)**. **Table 3** also shows the preferred choice of radiotherapy doses/fractions for individual metastases. Furthermore, the exact dose/fraction might be limited by the paracancerous tissue sites in the patient. Thus, based on the actual condition of the patient, we also considered adopting the preferred dose/fraction of RT, as shown in **Figure 2**.

**Table 3:** The priority order for the selection of disease sites.

Lesions	Prioritization	Preferred dose/fraction	Alternative dose fraction schedules
Primary lesions^a^	1	8 Gy/f	NA
Symptomatic vertebral lesions or symptomatic lesions adjacent to the spinal cord^b^	2	3 Gy/10f	8 Gy/f or 4Gy/5f
Vertebral body or disc metastasis lesions associated with the spinal cord or adjacent to the spinal cord	3	3 Gy/10f	8 Gy/f or 4Gy/5f
Symptomatic nonspinal bone metastatic lesions^c^	4	3 Gy/10f	8 Gy/f or 4Gy/5f
Lymph node lesion (patients with symptoms of compression)	5	4 Gy/5f	Conventional fractionation
Lymph node lesion (patients with no symptoms of compression)	6	4 Gy/5f	Conventional fractionation
Asymptomatic bone metastasis lesions	7	4 Gy/5f	8 Gy/f or 3 Gy/10f
Liver metastasis lesions	8	4 Gy/5f	NA
Lung metastasis lesions	9	4 Gy/5f	8 Gy/f or 3 Gy/10f
Other	10	According to the choice of the investigator and radiologist	According to the choice of the investigator and radiologist

The maximum number of metastases (per patient and/or per organ system) allowed for being eligible for the study was three disease sites. The disease sites were selected according to this prioritization order.

a In patients who did not receive treatment via radical prostatectomy and RT for the primary tumor, the primary lesions were given priority to receive RT.

b Patients who had pain in the vertebral section or disc metastasis lesions that were caused by spinal cord compression or adjacent to the spinal cord.

c Patients had pain due to nonspinal bone metastatic lesions that were caused by nonspinal cord compression (including thigh pain, scapula pain, etc.).

Although the dose/fraction of RT in this table was the preferred choice for disease sites, the exact dose/fraction was limited by the paracancerous tissues of the patient. Thus, according to the patient’s actual conditions, we also considered adopting the optional dose/fraction of RT in **Figure 2**.

Lymph nodes with a short diameter ≥1 cm on CT were considered metastatic lymph nodes. Moreover, pelvic wall, retroperitoneal, mediastinum, clavicle, and axilla lymph nodes were preferentially selected as gross tumor volume of lymph nodes (GTVnd) for RT (**Figure 2**) and without prophylactic irradiation of the lymph node drainage area. **Table 4** shows normal tissue dose constraints (33).

**Table 4:** Normal tissue dose constraints for stereotactic radiotherapy.

Description	Constraint	5 fractions (Gy)	
		Optimal	Mandatory
Heart	DMax (0.5 cm^3^)	<27	<27
Lungs	V20 Gy	–	<10%
Duodenum	DMax (0.5 cm^3^)	–	<35
	D10 cm^3^	–	<25
Stomach	DMax (0.5 cm^3^)	<33	<35
	D10 cm^3^	–	<25
Small bowel	DMax (0.5 cm^3^)	<30	<35
	D10 cm^3^	–	<25
Rectum	DMax (0.5 cm^3^)	–	<32
Liver	V10 Gy	<70%	–
Kidneys	Mean dose	<10	–
Bladder	D15 cm^3^	–	<18.3
	DMax (0.5 cm^3^)	–	<38
Brainstem (not medulla)	DMax (0.1 cm^3^)	<23	<31
Brain	D10 cm^3^	–	–

Normal tissue dose constraints were referred to as the “UK consensus on normal tissue dose constraints for stereotactic radiotherapy.”

DMax is the near-point maximum dose, referred to as D0.1 cm^3^ or D0.5 cm^3^, which was the minimum dose to the 0.1- or 0.5-cm^3^ volume of the organ receiving the highest doses; D10 cm^3^ and D15 cm^3^ were the minimum doses to the specified volume of the organ (10 or 15 cm^3^) that received the highest doses; V10 Gy or V20 Gy was the percentage volume of the organ receiving a dose of 10 or 20 Gy or higher.

Phase 3 was initiated 14 days after completing synchronous radiation, followed by tislelizumab maintenance therapy (200 mg; once every 3 weeks) until either the disease progressed or the patient developed unacceptable toxicity. However, notably, pseudoprogression might occur in the maintenance phase of tislelizumab, which would be required to be distinguished from actual progression by the researchers.

### Study Endpoints and Assessment

The primary endpoints included safety and ORR. ORR is defined as the proportion of patients who achieved a CR or partial response (PR). Secondary endpoints included the following indicators: prostate-specific antigen (PSA) response rate (PCWG3), disease control rate (DCR), OS, and progression-free survival (PFS) (radiographic PFS (rPFS), biochemical PFS (bPFS)). Here, rPFS is the time between the initial treatment start and radiographic PD, and bPFS is the time between the start of initial treatment and PD (caused by continuous elevation of PSA). We defined the PSA response rate as a 50% decline in PSA levels from baseline to 12 weeks after receiving tislelizumab monotherapy and the DCR as the proportion of patients whose best response was CR, PR, or stable disease (SD).

PFS is the time interval between therapy initiation and radiographic or biochemical PD, or death, whichever comes first. Furthermore, the exploratory purpose of this study was to explore the predictive biomarkers as described in the **Discussion** and **Appendix Table 1** that were related to efficacy and survival, which would help guide toward more individualized therapy.

In this study, laboratory testing and imaging examination were used to evaluate clinical symptoms, tumor response, adverse events (AEs), and biomarkers (**Appendix Table 1** **)**. A radiological review determined the tumor response every two treatment cycles (6 weeks). If disease progression was indicated based on imaging analysis, subsequent imaging analysis would be done to confirm this at least 4 weeks later. If pseudoprogression was confirmed, the investigator would then decide whether treatment could be continued.

The following data were recorded for safety and ORR assessment: demographics and medical history, physical examination, vital signs, laboratory testing, imaging examination, PSA, AEs, and biomarkers testing. The predictive biomarkers evaluated in this analysis included the following: the expression of DNA mismatch repair protein (MMR), androgen receptor splice variant 7 (AR-V7), tumor PD-L1, tumor-infiltrating lymphocyte (TIL) count, classification of immune cells and subsets (CD4^+^T, CD8^+^T, Treg, MDSC, M1-TAM (antitumor M1-like), M2-TAM (protumor M2-like)), the status of homologous recombination repair gene, AR pathway-related genes, and tumor mutation burden (TMB) level in tumors. In addition, we would also determine the classification of immune cells and subsets and TMB levels in peripheral blood.

### Follow-Up

Patients who successfully completed the interventional treatment were followed up for 30 days, and rAEs were recorded. In the case of no complications, patients were followed up every 2–3 months to collect antitumor treatment data and OS. However, patients who discontinued treatment for reasons other than PD were followed up every 8 weeks, followed up *via* imaging evaluation. If patients developed PD, they were followed up every 12 weeks to collect OS until death, consent to withdrawal, or the end of the study.

### Safety

During safety evaluation, we observed and recorded all AEs (including acute and late radiotherapy-related adverse events, immune-related adverse events (irAEs)), serious adverse events (SAEs), laboratory examination, general physical examination, performance status score, electrocardiogram, echocardiogram, thyroid function, myocardial markers, etc. The Common Toxicity Criteria for Adverse Events (CTCAE) v4.0 were used to classify AEs. Additionally, the radiation toxicity criteria of the Radiation Therapy Oncology Group (RTOG) and the European Organization for Research and Treatment of Cancer (EORTC) guidelines were used to assess the acute and late radiotherapy-related toxicities of grade and management (34). Secondly, the irAEs were also graded and managed according to the updated ASCO guidelines (35). Additionally, the “early” (<12 months) and “late” (>12 months) irAEs were categorized based on recent research data (36, 37). In this study, the following SAEs were considered: death, life-threatening AEs, in-patient or prolongation of existing hospitalization, permanent/severe disability, congenital anomalies/birth defects, or any significant medical event requiring intervention. Any AEs were registered during the AE reporting period. In addition, AEs associated with the investigational drug were also registered after reporting. All patients exhibiting SAEs were discontinued immediately, and the investigator reported cases to the sponsor as well as the ethics committee of the hospital within 24 h.

### Statistical Analysis

The sample size of this study was calculated according to Simon’s two-stage method (*α* = 0.05 (bilateral), *β* = 0.2) and by using efficacy as the estimation index. In a previous KEYNOTE-199 study, ORR was reported to be 5% in 133 patients who were PD-L1 positive in cohort 1 and 3% in 66 patients who were PD-L1 negative in cohort 2. The response rate of these 199 patients in the two cohorts was 4.5% (10). In this study, we hypothesized that the effective rate of radiotherapy combined with tislelizumab would reach 15%. Thus, 48 patients were enrolled and divided into two stages. A stage 1 study included 29 patients; stage 2 consisted of 15 patients when the ORR from stage 1 reached at least 1 (RECIST v1.1). Four patients were then added, considering a 10% loss to follow-up or dropout rate.

The primary efficiency analysis will be performed on the complete analysis set, including all subjects assigned to interventional therapy. Patients who received ≥1 dose of the investigational drug and recorded safety indicators were evaluated for safety analysis. Descriptive statistics were provided using medians (ranges) and means (standard deviations) for continuous variables and frequency (proportions) for categorical variables. The Clopper–Pearson method was used for PSA response rates and 95% CI. The Kaplan–Meier method was used to estimate the PFS and OS; the median values were estimated with a 95% CI. All statistical analyses were two-sided, and *p* < 0.05 was considered significant. All statistical analyses were done using SPSS software v25.0.

### Data Collection and Management

All researchers in this study were responsible for the accuracy of the collected data as well as data management. The data monitoring committee (DMC) conducted regular data monitoring during and after the study.

## Discussion

This study presents the first investigational analysis of the safety and efficacy of tislelizumab combined multisite RT for patients with mCRPC who had experienced failed ADT and at least one second-line endocrine therapy failure. Until now, the poor responses of immunotherapy against PCa might be attributed to its characteristics of low immune infiltration, low tumor mutation load, and low antigen presentation (38). Additionally, PCa evades and inhibits antitumor immunity *via* elevated expression of PD-L1 and enrichment of Tregs in both tumor and peripheral blood [19-21]. Interestingly, various studies have confirmed that a combination of immunotherapy and RT could constitute a promising strategy for the synergistic enhancement of treatment efficacy. In the last few years, several studies on various types of tumors have explored the combination of radiotherapy and immunotherapy, such as breast cancer, melanoma, nonsmall-cell lung cancer (NSCLC), and esophageal squamous cell carcinoma (39). All studies showed promising antitumor activity and acceptable tolerability. In recent years, there have been significant advances in the treatment of PCa, and several new treatment strategies for mCRPC with clinically proven survival benefits for mCRPC have been developed (10, 11, 40). However, there is still a lack of appropriate strategies for patients with mCRPC who have experienced ADT failure and second-line endocrine therapy. A recent study revealed that avelumab with SABR showed promising activity and acceptable toxicity in treatment-refractory mCRPC (26), indicating that immunotherapy combined with RT was still the best area of research. However, the data were limited to only one combination of tislelizumab and RT, limiting the treatment potential of mCRPC. Therefore, the combination treatment of tislelizumab plus multisite radiotherapy represents a potential approach and needs further investigation for patients with mCRPC who had experienced failure of ADT and second-line endocrine therapy.

The present study has been designed for three treatment phases. Tislelizumab monotherapy aims to observe the efficacy of tislelizumab monotherapy for patients with mCRPC by measuring changes in patients’ PSA levels and symptoms. Due to the “cold tumor” characteristics of PCa, we predict that the 2-cycle efficacy of tislelizumab monotherapy may be insignificant. But it may show effectiveness in some patients who might benefit from immunotherapy in a short period, and those patients are worth being screened for biomarkers for immunomonotherapy.

Some patients with an immediate immune reaction to immunotherapy may result in irAEs. Previous studies reported that patients who experienced irAEs demonstrated marked improvements in immunotherapy efficacy compared to those with low toxicity (41). However, if irAEs occur prematurely (≤8 weeks), immunotherapy is likely to be discontinued due to toxicity. Therefore, we designed two cycles of tislelizumab monotherapy. Furthermore, recent retrospective studies have indicated that “early” irAEs were associated with poor prognosis, and the immunosuppressive treatment for irAEs may hinder anti–PD-1 monotherapy efficacy (42, 43). Therefore, the monotherapy phase would also help understand whether the early irAEs will occur in tislelizumab monotherapy, thus assessing the safety of tislelizumab monotherapy. Followed by the tislelizumab combined RT, comparing the efficacy and safety of tislelizumab monotherapy, the safety and synergistic effect of multisite RT combined with immunotherapy can be better observed. Furthermore, the safety and efficacy of RT can still be fully observed due to a delayed effect from RT, even if entering the tislelizumab monotherapy maintenance phase. Notably, suppose patients with mCRPC obtain a good survival benefit from this study, then the treatment value of tislelizumab as a maintenance therapy method in these patients could obtain preliminary verification.

Until now, there has been a lack of consensus regarding the ideal dose of RT in combination with immunotherapy. SBRT, as a novel RT method, is essential in the treatment of early primary cancer and oligometastatic disease, such as oligometastatic (≤5 lesions) PCa, early-stage nonsmall-cell lung cancer, and liver cancer (44, 45). It has the potential to deliver a small amount of ultra-high doses of radiation to relatively small target lesions, achieving local control with a low risk of toxicity (46). For advanced cancer patients with multiple metastases, the dose of irradiated lesions might be different to achieve excellent local control with a low risk of toxicity and more potent immune activation effects. Therefore, individualized RT will be performed in this study. We will still preferentially select treatment with SBRT, 40 Gy in five fractions, every other day for primary lesions.

On the one hand, the hypofractionated SBRT regimen of 40 Gy/5 is delivered to accommodate the radiation tolerance of organs at risk. On the other hand, the hypofractionated SBRT regimen facilitates immunogenic cell death (ICD), leading to the release of tumor antigens, thus amplifying the efficacy of immunotherapy (47). However, the majority of patients with PCa usually present with multiple distant metastases. In such cases, the dose regimens, guidelines, and normal tissue constraints determined in carefully conducted, high-quality prospective trials should be adopted (44). According to the ASTRO guidelines and the SABR-COMET study (48), 30 Gy in 10 fractions was preferred to treat bone metastases in the present study, which plays a role in palliative pain relief and modulates the immune response microenvironment (49). For liver metastases, 20 Gy in 5 fractions was standard institutional practice. According to the 2011 consensus guidelines, the radiation dose fractionation for lung metastases mainly included 8 Gy in 1 fraction, 20 Gy in 5 fractions, and 30 Gy in 10 fractions (50), and 20 Gy in 5 fractions was preferred for the treatment of bone metastases in the present study.

Furthermore, the present study combined immunotherapy treatment for patients with mCRPC who had failed multiline therapy and had a relatively long survival time. Thus, irAEs are important safety parameters to consider, especially for fatal irAEs such as pneumonitis, neurologic toxicity, colitis/diarrhea, and hepatitis (51) as the significant life-threatening factors for elderly patients. Using the combination of higher radiation doses with anti-PD-1 immunotherapy may cause irAEs to occur in the long term. Therefore, SBRT with relatively low radiation doses was performed based on security considerations in this study.

Tislelizumab is a novel IgG4 anti-PD-1 mAb monoclonal antibody that minimizes binding to FcγR on the surface of macrophages to eliminate antibody-dependent phagocytosis, resulting in a higher affinity for PD-1 compared with pembrolizumab and nivolumab (12). Both clinical literature and pharmacokinetics (PK) analysis have demonstrated that tislelizumab is well tolerated for multiple advanced tumor types and supports fixed dosing (200 mg) (52). Therefore, in the present study, we used a fixed-dose instead of dose-escalation exploration, which avoided the uncertainty caused by dose exploration and improved the effectiveness of this study.

Additionally, the most important aim was to maximize the therapeutic benefits by developing predictive biomarkers of immunotherapy responsiveness. Several biomarkers have been associated with the treatment effect of anti-PD-1 therapy, such as TMB, mismatch repair deficiency (dMMR), PD-1 expression, and TIL number (53). At the same time, these have been reported to be relatively rare in patients with mCRPC. TMB, a biomarker independent of PD-L1 expression, has been revealed to have a significant association with ORR across multiple cancer types (54). However, the application of TMB in mCRPC needs further validation. A previous study suggested that tumors with dMMR are susceptible to PD-1 and PD-L1 inhibitors.

Meanwhile, dMMR tumors exhibit a dense infiltration of CD^8+^ TILs that have been shown to induce a better and more durable response (55). Several clinical studies have indicated the association between dMMR and immunotherapy-related responses and better prognosis in other solid tumors (55–57). However, this correlation needs further exploration in mCRPC. Numerous clinical trials have investigated that PD-L1 expression is the most widely adopted predictor, and high PD-L1 expression is associated with clinical benefit and response rate improvement in anti-PD-1/anti-PD-L1 therapy (53). TIL is a vital component that influences the tumor immune microenvironment and is used for the prediction of immunotherapy combined with the expression of PD-L1 expression. Elevated levels of baseline TIL and PD-L1 expression in breast cancers were found to be associated with an increased probability of pathologic complete response (58). However, in the immunotherapy combination of multisite RT for mCRPC treatment, the predictive value of PD-L1 expression and TIL counts is vague and deserves further investigation.

Furthermore, studies have demonstrated that genomic alterations might elicit a broad impact on the tumor microenvironment, contributing to the promotion and maintenance of responses to immunotherapy (59–61). Thus, a genomic analysis needs to be performed in this study to determine the impact of genomic alterations (such as mutations in the exonuclease domain of the DNA polymerase epsilon (POLE), high tumor mutational burden, and the presence of biallelic loss of CDK12, among others) on immunotherapy for PCa, for the early detection and identification of novel therapeutic targets. Thus, it would be crucial to establish a comprehensive assessment framework involving multiple biomarkers for interrogating the tumor immune landscape and selecting sensitive patients.

However, this study has several limitations. It is a nonrandomized study with a small sample size. Therefore, the results of this study would provide preliminary support for future randomized, controlled trials to assess the combined therapeutic regimen for patients with mCRPC.

Thus, this study is the first attempt to evaluate the efficacy and safety of tislelizumab with multisite radiotherapy for patients with mCRPC who have failed ADT and second-line endocrine therapy, in an attempt to provide an accurate and effective combined treatment for patients with mCRPC and improve the survival status of patients.

## Data Availability Statement

The original contributions presented in the study are included in the article/**Supplementary Material**. Further inquiries can be directed to the corresponding author.

## Ethics Statement

The studies involving human participants were reviewed and approved by Ethics Review Committee of West China Hospital, Sichuan University (Ethical approval number: 2021203). The patients/participants provided their written informed consent to participate in this study.

## Author Contributions

KC, YQW, YC, YCW, and ZL were involved in the study conception and design. ZL provided administrative support. KC, YQW, and YC provided materials and samples. KC, JZ, and XQ participated in data collection. KC, YQW, YC, YCW, ZL, JZ, and XH contributed to analysis and interpretation of the data and wrote the manuscript. All authors were involved in the final approval of the manuscript. All authors agreed to be accountable for all aspects of the work and approved the final manuscript.

## Funding

This study was partly supported by the Sichuan Science and Technology Department Key Research and Development Project (22YFS0336) and 1.3.5 project for disciplines of excellence, West China Hospital, Sichuan University (ZYJC18048).

## Conflict of Interest

The authors declare that the research was conducted in the absence of any commercial or financial relationships that could be construed as a potential conflict of interest.

## Publisher’s Note

All claims expressed in this article are solely those of the authors and do not necessarily represent those of their affiliated organizations, or those of the publisher, the editors and the reviewers. Any product that may be evaluated in this article, or claim that may be made by its manufacturer, is not guaranteed or endorsed by the publisher.

## Supplementary Material

Supplementary Table 1 Assessment timetable. In peripheral blood, the biomarkers and the main detection techniques included immune cells and subsets, such as CD^4+^T/CD^8+^T/Treg/MDSC/M1-TAM/M2-TAM (FCM); TMB (NGS). *PSA*, prostate-specific antigen; *AEs*, adverse events; *SAEs*, serious adverse events; *MMR*, mismatch repair protein; *PD-1*, programmed death-1; *PD-L1*, programmed death-ligand 1; *PD-L2*, programmed death-ligand 2; *TILs*, tumor-infiltrating lymphocytes; *MDSCs*, myeloid-derived suppressor cells; *M1-TAM*, antitumor M1-like; *M2-TAM*, protumor M2-like; *LAG-3*, lymphocyte activation gene 3; *TIM-3*, T-cell immunoglobulin mucin-3; *AR-V7*, androgen receptor variant 7; *TMB*, tumor mutation burden. Biomarker detection techniques: *IHC*, immunohistochemical; *HE staining*, hematoxylin-eosin staining; *FCM*, flow cytometry; *RNA-seq*, RNA sequencing. ^a^In a tumor, the biomarkers and the main detection techniques were as follows: MMR (IHC), PD-1/PD-L1/PD-L2 (IHC), TIL (HE staining and IHC), immune cells, and subsets, such as CD^4+^T/CD^8+^T/Treg/MDSC/M1-TAM/M2-TAM (FCM); costimulatory factor: LAG-3/TIM-3/CD28/CD80/CD137 (GenecastPlex-59 panel), AR-V7 (IHC and NGS), homologous recombination repair genes (NGS), AR pathway-related genes (NGS), and RNA (RNA-seq).

## Abbreviations

mCRPC, metastatic castration-resistant prostate cancer; ADT, androgen deprivation therapy; PSA, prostate-specific antigen; rPFS, progression-free survival; bPFS, biochemical progression-free survival; PCa, prostate cancer; PD-1, programmed death receptors; PD-L1, programmed death-ligand 1; CR, complete response; RT, radiotherapy; SBRT, stereotactic body radiotherapy; PD, disease progression; PCWG3, Prostate Cancer Working Group 3; OS, overall survival; IMRT, intension-modulated radiotherapy; CT, computed tomographic; ORR, objective response rate; PR, partial response; PSA, prostate-specific antigen; PFS, progression-free survival; SD, stable disease; AEs, adverse events; MMR, mismatch repair protein; AR-V7, androgen receptor splice variant 7; TIL, tumor-infiltrating lymphocyte; M1-TAM, antitumor M1-like; M2-TAM, protumor M2-like; TMB, tumor mutation burden; TC, T cell receptor; irAEs, immune-related adverse events; SAEs, serious adverse events; CTCAE, Common Toxicity Criteria for Adverse Events; NSCLC, nonsmall-cell lung cancer; ICD, immunogenic cell death; dMMR, mismatch repair deficiency; POLE, polymerase epsilon.
