# Supplementary material for: Multisite Radiotherapy Combined With Tislelizumab for Metastatic Castration-Resistant Prostate Cancer With Second-Line and Above Therapy Failure: Study Protocol for an Open-Label, Single-Arm, Phase Ib/II Study
Source: Front Oncol. 2022 Jul 7;12:888707. doi: 10.3389/fonc.2022.888707 (PMC9300836; doi:10.3389/fonc.2022.888707)
Supplement: Supplementary Table 1 — Assessment timetable. In peripheral blood, the biomarkers and the main detection techniques included immune cells and subsets, such as CD4+T/CD8+T/Treg/MDSC/M1-TAM/M2-TAM (FCM); TMB (NGS). PSA, prostate-specific antigen; AEs, adverse events; SAEs, serious adverse events; MMR, mismatch repair protein; PD-1, programmed death-1; PD-L1, programmed death-ligand 1; PD-L2, programmed death-ligand 2; TILs, tumor-infiltrating lymphocytes; MDSCs, myeloid-derived suppressor cells; M1-TAM, antitumor M1-like; M2-TAM, protumor M2-like; LAG-3, lymphocyte activation gene 3; TIM-3, T-cell immunoglobulin mucin-3; AR-V7, androgen receptor variant 7; TMB, tumor mutation burden. Biomarker detection techniques: IHC, immunohistochemical; HE staining, hematoxylin-eosin staining; FCM, flow cytometry; RNA-seq, RNA sequencing. aIn a tumor, the biomarkers and the main detection techniques were as follows: MMR (IHC), PD-1/PD-L1/PD-L2 (IHC), TIL (HE staining and IHC), immune cells, and subsets, such as CD4+T/CD8+T/Treg/MDSC/M1-TAM/M2-TAM (FCM); costimulatory factor: LAG-3/TIM-3/CD28/CD80/CD137 (GenecastPlex-59 panel), AR-V7 (IHC and NGS), homologous recombination repair genes (NGS), AR pathway-related genes (NGS), and RNA (RNA-seq). [file Table_1.docx]

**Appendix Table 1. Assessment timetable**

| Project | | Screening | Treatment | | | | Study end | Follow up |
| --- | --- | --- | --- | --- | --- | --- | --- | --- |
|  |  |  | Start of each cycle (within 1 week before) | End of the induction therapy (every 2 cycles) | End of the combination therapy | End of the maintenance therapy |  |  |
| Informated consent | | **√** |  |  |  |  |  |  |
| Demographics | | **√** |  |  |  |  |  |  |
| Past medical history | | **√** |  |  |  |  |  |  |
| Height and weight | | **√** | √ |  |  |  | √ | √ |
| Vital signs | | **√** | **√** | **√** | **√** | **√** | √ | √ |
| ECOG score | | **√** | **√** | **√** | **√** | **√** | √ | √ |
| Physical examination | | **√** | **√** | **√** | **√** | **√** | √ | √ |
| PSA | | **√** | **√** | **√** | **√** | **√** | √ | √ |
| Laboratory testing | | **√** | **√** | **√** | **√** | **√** | √ | √ |
| Imaging examination | | **√** |  | **√** | **√** | √ | √ | √ |
| Toxicity evaluation | |  | **√** | **√** | **√** | **√** |  |  |
| AEs and SAEs | |  | **√** | **√** | **√** | **√** | √ | √ |
| Biomarkers  detection* | tumor | **√** |  |  |  |  |  |  |
|  | peripheral blood | **√** |  | **√** | **√** |  |  |  |

*In tumor, the biomarkers and the main detection techniques were as follows: MMR (IHC), PD-1/ PD-L1/ PD-L2 (IHC), TIL (HE staining and IHC), immune cells and subsets, such as CD^4+^T/CD^8+^T/Treg/MDSC/M1-TAM/M2-TAM (FCM), co-stimulatory factor: LAG-3/TIM-3/CD28/CD80/CD137 (GenecastPlex-59 panel), AR-V7 (IHC and NGS), homologous recombination repair genes (NGS), AR pathway related genes (NGS), and RNA (RNA-seq).

In peripheral blood, the biomarkers and the main detection techniques included immune cells and subsets, such as CD^4+^T/CD^8+^T/Treg/MDSC/M1-TAM/M2-TAM (FCM); TMB (NGS).

PSA, prostate-specific antigen; AEs, adverse events; SAEs, serious adverse events. MMR, mismatch repair protein; PD-1, programmed Death-1; PD-L1, programmed death ligand 1, PD-L2, programmed death ligand 2; TIL, tumor infiltrating lymphocytes; MDSC, myeloid-derived suppressor cells; M1-TAM, antitumor M1-like; M2-TAM, pro-tumor M2-like; LAG-3, lymphocyte activation gene 3; TIM-3, T-cell immunoglobulin mucin-3; AR-V7, androgen receptor variant 7; TMB, tumor mutation burden. Biomarkers detection techniques: IHC, immunohistochemical; HE staining, hematoxylin-eosin staining; FCM, flow cytometry; RNA-seq, RNA sequencing.
